# Selective inhibition of aggregation/fibrillation of bovine serum albumin by osmolytes: Mechanistic and energetics insights

**DOI:** 10.1371/journal.pone.0172208

**Published:** 2017-02-16

**Authors:** Moumita Dasgupta, Nand Kishore

**Affiliations:** Department of Chemistry, Indian Institute of Technology Bombay, Powai, Mumbai, India; Russian Academy of Medical Sciences, RUSSIAN FEDERATION

## Abstract

Bovine serum albumin (BSA) is an important transport protein of the blood and its aggregation/fibrillation would adversely affect its transport ability leading to metabolic disorder. Therefore, understanding the mechanism of fibrillation/aggregation of BSA and design of suitable inhibitor molecules for stabilizing its native conformation, are of utmost importance. The qualitative and quantitative aspects of the effect of osmolytes (proline, hydroxyproline, glycine betaine, sarcosine and sorbitol) on heat induced aggregation/fibrillation of BSA at physiological pH (pH 7.4) have been studied employing a combination of fluorescence spectroscopy, Rayleigh scattering, isothermal titration calorimetry (ITC), dynamic light scattering (DLS) and transmission electron microscopy (TEM). Formation of fibrils by BSA under the given conditions was confirmed from increase in fluorescence emission intensities of Thioflavin T over a time period of 600 minutes and TEM images. Absence of change in fluorescence emission intensities of 8-Anilinonaphthalene-1-sulfonic acid (ANS) in presence of native and aggregated BSA signify the absence of any amorphous aggregates. ITC results have provided important insights on the energetics of interaction of these osmolytes with different stages of the fibrillar aggregates of BSA, thereby suggesting the possible modes/mechanism of inhibition of BSA fibrillation by these osmolytes. The heats of interaction of the osmolytes with different stages of fibrillation of BSA do not follow a trend, suggesting that the interactions of stages of BSA aggregates are osmolyte specific. Among the osmolytes used here, we found glycine betaine to be supporting and promoting the aggregation process while hydroxyproline to be maximally efficient in suppressing the fibrillation process of BSA, followed by sorbitol, sarcosine and proline in the following order of their decreasing potency: Hydroxyproline> Sorbitol> Sarcosine> Proline> Glycine betaine.

## Introduction

The aggregation of proteins can take place under various environmental conditions, giving rise to higher order supramolecular structures including fibrillar forms [1,2]. The phenomenon could be either, temperature, pH or concentration driven, or combination of these said factors. The major pathways of protein aggregation are–(i) through the partially folded states or intermediates, (ii) self association of protein molecules, (iii) chemical linkages or via chemical degradation [1]. The rationale behind the aggregation pathways are the net surface charge that could be repelling as well as the hydrophobic effect which gives rise to attractive forces between the monomers [3]. In case, where the aggregation occurs due to high temperature, the hydrophobic interactions play significant role rather than electrostatic interactions [4]. Again at a particular temperature and pH, the concentration determines the size of the aggregates and variation of concentration under such conditions could bring about formation of oligomers to gel from those aggregates. The aggregation pathways are complex enough to give rise to the formation of aggregates of diverse morphologies based on solution conditions and above mentioned physical factors [3]. Amyloid fibrils are categorized into one of those morphologies and are associated with neurodegenerative diseases, while the prefibrillar aggregates are the causative agents of cytotoxic effects *in vivo* [2,3]. Structurally, the protein aggregates are found to be β-sheet rich, where amyloids are typical cross β structures that are aligned perpendicularly to the axis of the fibril [3]. However, in recent time, active research is going on, in order to implement these fibrillar structures, generated *in vitro*, in the field of biomedicine and bionanotechnology [5]. These fibrils find their application from development of peptide nanotubes useful in bio-electrochemical sensor applications to creation of scaffolds applicable in tissue engineering and drug delivery [4]. The fibrils could also be used for bacterial biofilm development [2].

However, inhibition of protein aggregation in vivo, is extremely important in order to prevent and control the occurrence of neurodegenerative diseases. Herein lies the importance of studying the effect of external small molecules, that could potentially arrest the aggregation propensity of proteins and stabilize the native form. Our study can guide the development of therapeutic strategies against the diseases caused due to protein aggregation/fibrillation. The naturally occurring small molecules, called osmolytes, synthesized in various organisms in response to stress [6], are known to play crucial role in protecting cell against high osmotic condition by maintaining cell volume and fluid balance as well as increasing the thermodynamic stability of protein molecules thereby stabilizing the functional folded form. Hence the osmolytes are also known as “chemical chaperones” [6,7]. These organic compounds could be amino acids (e.g. proline, glycine and arginine), various polyols (e.g. sorbitol and sucrose) and methyl amines (e.g. betaine and trimethylamine-N-oxide) [6]. There are several reports on osmolyte mediated prevention of protein misfolding and aggregation [8–10]. The enhancement in thermal stability of proteins, brought about by the osmolytes, is known to be via the phenomenon of preferential hydration [8,9,11–13].

**Fig 1:**
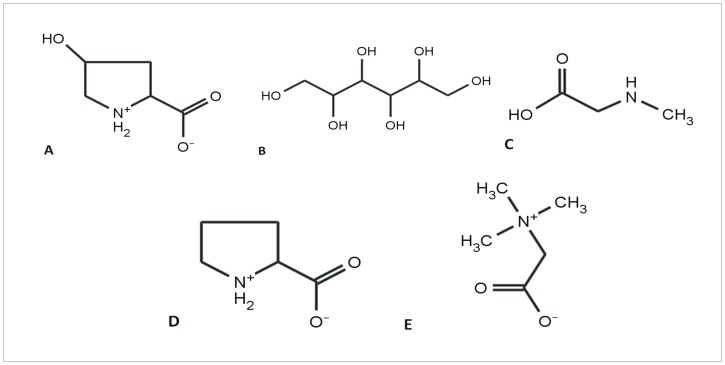
Chemical structures of osmolytes. Chemical structures of (A) hydroxyproline, (B) sorbitol, (C) sarcosine, (D) proline and (E) glycine betaine.

In the current study, an approach has been taken to explore and understand the aggregation behavior of bovine serum albumin (BSA) at physiological pH conditions (pH 7.4), in the absence and presence of various osmolytes (proline, hydroxyproline, glycine betaine, sarcosine and sorbitol), employing a combination of spectroscopic, calorimetric, light scattering and imaging techniques. The aim is to understand the mode of interaction of these osmolytes with different stages of BSA aggregates which can ultimately aid in design and development of suitable inhibitors/drugs to arrest such protein aggregation phenomenon thereby preventing the associated diseases. The chemical structures of these osmolytes are shown in Fig 1.

Serum albumin is the transport protein of the blood circulatory system that bind and hence act as a carrier for a wide variety of exo- and endogenous ligands [14]. BSA is made up of a single polypeptide chain consisting of 583 amino acids, arranged into three homologous domains which is primarily made up of alpha helices as the secondary structure with some turns and random coils as well [2]. There are a few reports on aggregation and fibrillation behavior of BSA under various conditions of temperature and concentration. One such being the study carried out by Holm et. al [2], where they observed a 40% increase in β-sheet structure upon aggregation, while retaining significant α-helical structures as well and these also lack a protease-resistant core and are not cytotoxic. There are 17 disulfide bonds in a BSA monomer with Cys 34 being unpaired and this cysteine residue has been shown to affect the aggregation rate of BSA [15]. Glycation of globular albumin can induce these proteins to adopt cross-β structure thereby giving rise to amyloid fibrils [16]. Therefore, aggregation of bovine serum albumin is expected to adversely affect its transport ability including drug transport, thereby leading to several metabolic disorder.

## Materials and methods

### Materials

Bovine serum albumin (≥ 98%), thioflavin T (Dye content: 65–75%), 8-anilinonaphthalene-1-sulfonic acid (ANS) (> 99%), L-proline (> 99%), 4-hydroxy-trans-L-proline (> 99%), glycine betaine (≥ 99%), sarcosine (> 98%) and D-sorbitol (> 98%) of the best purity grade were obtained from Sigma Aldrich Chemical Company, USA. The purities of all the chemicals used have been mentioned in the parenthesis. The protein and other chemicals were dissolved in 20 mM phosphate buffer containing 100 mM NaCl and pH adjusted to 7.4. Double distilled water was used for preparation of the buffer. The BSA dissolved in buffer was centrifuged after 2 hours followed by determination of concentration of the protein stock solution. The concentration of BSA stock solution, ThT and ANS were determined on Jasco V-550 UV-visible double beam spectrophotometer using the absorbance A_280_^1%^ = 6.8 [17] for BSA and the extinction coefficients *ε*_416_ = 26,620 M^-1^cm^-1^ [18] and *ε*_350_ = 5000 M^-1^cm^−1^ [19], ThT and ANS respectively.

### Methods

#### UV–visible spectroscopy

Thermal denaturation of BSA was carried out on Jasco V-550 spectrophotometer to obtain the thermal transition temperature (T_m_). The protein concentration was kept fixed at 15.06 μM. The absorbance was measured at different temperatures from 298 K to 359 K and at a fixed wavelength [20] of 293 nm. A Cole–Parmer Polystat temperature controller unit with an accuracy of T = ±0.1 K was used to control the temperature around the cuvettes. The value of T_m_ was obtained after analysis by EXAM programme of Kirchhoff [21].

#### Fluorescence spectroscopy

**ThT binding kinetics**: The kinetics of fibrillation of 75.30 μM BSA was monitored in real time, from the binding of amyloid specific dye [22] thioflavin T (ThT) (50 μM) on a Cary Eclipse spectrofluorimeter (Varian, USA) in quartz cuvette of 1 cm path length. ThT, a cationic benzothiazole dye, is a well established marker of amyloid fibrils [22,23]. The excitation and emission slit widths were fixed at 5 nm each. The excitation and recording of emission of ThT were done at 450 nm and at 485 nm respectively [24,25] as a function of time to monitor the progress of aggregation, in the presence and absence of different concentrations of various osmolytes. The reported fluorescence emission spectra of the complexes were corrected by subtracting the reference spectra of the control solutions containing same amount of the dye.

**Binding of ANS**: Steady state fluorescence emission measurements of 50 μM of 8-Anilinonaphthalene-1-sulfonic acid (ANS) with the BSA aggregates formed in the presence and absence of different concentrations of various osmolytes were monitored, on the Cary Eclipse spectrofluorimeter (Varian, USA) in quartz cuvette of 1 cm path length. The excitation wavelength for all the ANS binding experiments was 365 nm with the excitation and emission slit widths being fixed at 5 nm each. The background emission spectra due to BSA aggregates and buffer were subtracted from the spectra of the solutions of the different stages of BSA aggregates in the absence of any osmolyte while in the presence of the osmolytes, subtraction of background emission spectra due to the BSA aggregates, osmolytes and buffer were carried out from the respective sample readings.

#### Rayleigh scattering

The Rayleigh scattering experiments were performed on a Cary Eclipse spectrofluorimeter (Varian, USA) in quartz cuvette of 1 cm path length, keeping the wavelength of excitation and emission fixed at 350 nm. The intensity of Rayleigh Scattering was measured for a duration of 600 minutes at 333.15 K, in the absence and presence of different concentrations of the osmolytes. Prior to all the experiments, the buffer was degassed to avoid scattering due to bubbles. The concentration of the protein was kept constant at 75.30 μM with the excitation and emission slit widths being fixed at 2.5 nm for all the Rayleigh Scattering measurements. The reported intensities were corrected for those of buffer/ osmolyte solutions under similar conditions.

#### Isothermal Titration Calorimetry

The ITC experiments were designed and carried out on Nano ITC (TA Instruments, New Castle, DE, USA) to understand the nature of interactions of these osmolytes with those of the BSA aggregates. The solutions of different stages of BSA aggregates were titrated into 0.50 M of each of the osmolytes, at 298.15 K. The reference cell of ITC was filled with dialysate phosphate buffer (pH 7.4), while 0.50 M of osmolyte solution (prepared in the dialysate buffer) was loaded into the sample cell. A 250 μl injection syringe was filled with 75.30 μM BSA aggregate. Separate fillings of the injection syringe were done for different stages of the aggregates and hence separate experiments were carried with each of the stages of aggregates thus formed as well as for each of the osmolytes. During the titration, aliquots of 10 μl of the solution of aggregates were injected into the sample cell containing the osmolyte, at an interval of 5 minutes between the successive injections. A total of 25 injections, each lasting for 20s, were made from the computer controlled syringe. Stirring of the solution in the sample cell was done at a speed of 250 rpm. For each experiment, corresponding aggregate and osmolyte dilutions were carried out and then subtracted from the main experiment, to obtain the heat of interaction between the aggregates and the particular osmolyte solution. Separate experiments were also done, at 298.15 K, to measure the heat of dilution of the native protein as well as different stages of aggregation, in absence of any osmolyte. Nano Analyzer software was used for the purpose of data analysis.

#### Dynamic Light Scattering (DLS)

A 90 Plus Particle Size Analyzer from Brookhaven Instruments Corp. was used for performing the light scattering experiments at 298.15 K with the aggregates formed from 75.30 μM of BSA after incubation at 333.15 K for about 600 minutes, in the presence and absence of some of the osmolytes. Small particles in suspension undergo random thermal motion known as Brownian motion. This random motion is modeled by the Stokes-Einstein equation, which is given below and used for particle size analysis.

$$D=k_{B}T/3\pi \eta d$$ (1)

Where, *D* is the diffusion coefficient at infinite dilution, *k*_*B*_ is Boltzman constant (1.3807 x 10^−23^ J/K), *T* is the temperature in Kelvin, *η* is the viscosity of solvent and *d* is hydrodynamic diameter of the particles [26].

#### Transmission Electron Microscopy (TEM)

The TEM images of BSA aggregates, formed in the presence and absence osmolyte were collected using JEM 2100F JEOL-Field Emission electron microscope and JEM 2100 JEOL HRTEM, both operating under an accelerating voltage of 200 kV. The protein samples (75.30 μM) in absence and presence of 1.00 M solution of the osmolytes, were incubated at 333.15 K, for about 600 minutes, under stirring condition. Thereafter, they were loaded onto Formvar-coated 300 mesh copper grids and dried before collecting the images.

## Results and discussion

### Determination of T_m_ and temperature dependent aggregation kinetics of BSA

**Fig 2:**
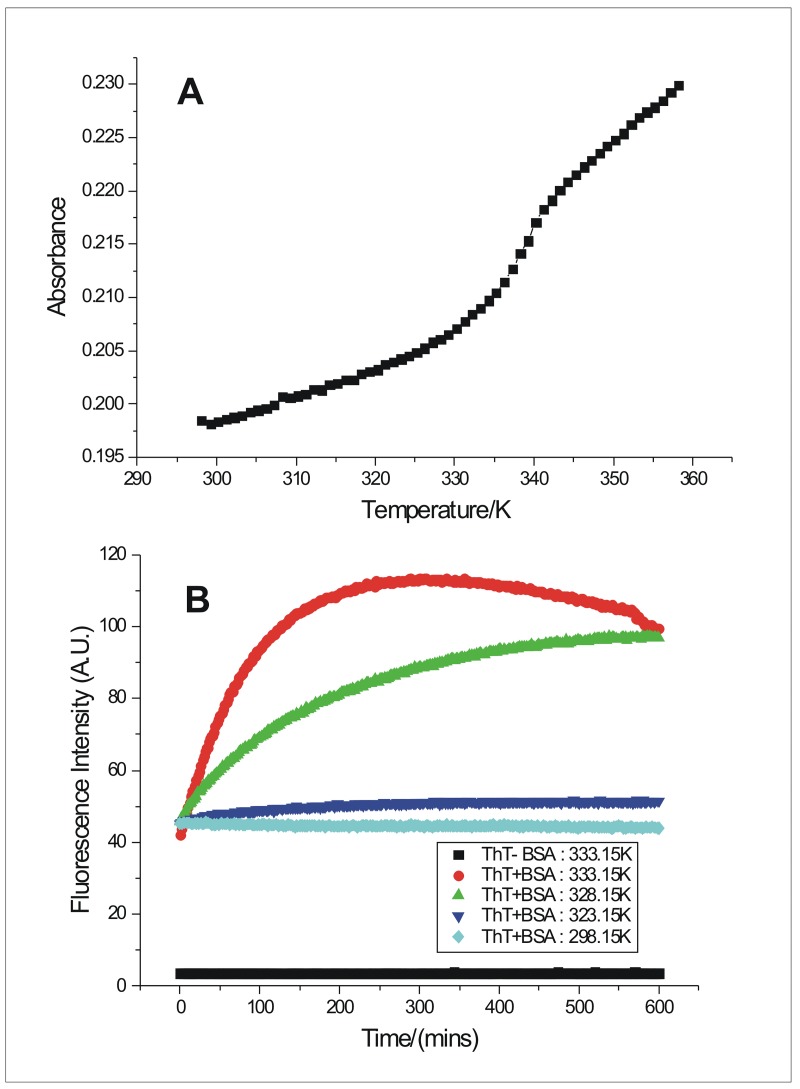
Thermal denaturation of BSA and temperature dependent kinetics of BSA fibrillation. (A) Representative plot of absorbance versus wavelength for thermal denaturation of 15.06 μM BSA and (B) Temperature dependent kinetics of 75.30 μM BSA fibrillation monitored from ThT fluorescence emission at pH 7.4.

The thermal transition temperature, T_m_ obtained from the thermal denaturation experiments was (334.4±0.1) K (Fig 2A). To check the reversibility of the denaturation process the thermally denatured BSA solution was slowly cooled and again heated under similar conditions with simultaneous monitoring of the absorbance of the solution at 293 nm. The process was observed to be completely irreversible signifying the formation of more stable structure upon fibrillation compared to the native state. In order to form aggregates, access of the partially denatured/unfolded states of the protein would be required and those states are generally present at temperatures around T_m_ [2]. Temperature dependent ThT binding kinetics experiments were carried out to monitor the fibrillation of BSA at temperatures 298.15 K, 323.15 K, 328.15 K and 333.15 K. There was no fluorescence emission from ThT in buffer, in the absence of any protein (Fig 2B), while in the presence of BSA at 298.15 K, there was a non-zero fluorescence emission intensity of ThT, which remained constant over the entire time period of 600 minutes. This indicates the occurrence of some interaction of ThT molecules with BSA (pH 7.4) at 298.15 K, providing evidence for presence of ThT binding sites on BSA molecules. ThT has been reported to interact with a wide variety of non-fibrillar molecules as well, such as DNA [27], mucopolysaccharides, connective tissues like cartilage matrix, elastic fibers [20,28] and even native α-helical protein such as serum albumins [29]. The absence of any increase in the fluorescence emission of ThT with time signifies absence of any fibrillation taking place at 298.15 K over the period of 600 minutes. At 323.15 K, there is a slight increase in the ThT fluorescence emission intensity compared to that at 298.15 K, denoting only a small amount fibrillation taking place over the mentioned time period. The ThT fluorescence emission data (Fig 2B) obtained at temperatures 328.15 K and 333.15 K, show the occurrence of significant fibrillation of BSA, with a faster rate of aggregation at 333.15 K. Therefore, the temperature of fibrillation of BSA used, for all the subsequent experiments in our work, was 333.15 K.

The ThT binding kinetics data obtained at 328.15 K and 333.15 K, show that fibrillation mechanism of BSA does not include a lag phase and is consistent with the reports [2, 3, 30] on lag phase/nucleation phase independent aggregation mechanism of BSA (see Fig 2B). Absence of lag phase in the fibrillation process, has been observed for other proteins as well, such as acylphosphatase [31], serum albumins (HSA, PSA, SSA, RSA) [32, 33], calcium- and zinc-binding protein S100A6 under acidic conditions [34], in vivo amyloid fibril formation by ^HF488^Aβ(1–42) peptide [35], heat induced protofibril formation by barstar [36], etc. Absence of lag/nucleation phase of the kinetic curves of BSA fibrillation suggests that its fibrillation process follows the mechanism of downhill polymerization [37, 38].

To address the absence of lag phase in the fibrillation kinetics of BSA, the role of charge-charge repulsion on surface of the protein molecules needs to be discussed. Electrostatic repulsions between like charges on the surface of the protein molecules will hinder their association into oligomeric structures thereby reducing the rate of aggregation [39–41]. Therefore, the presence of high concentration of NaCl in the buffer of pH 7.4 for preparation of BSA (pI of BSA is 4.7 [42]) minimizes the repulsion between the surface charges on BSA molecules thereby aiding the aggregation process of BSA by rapid rate of its association. This phenomenon of screening of like charges on protein molecules is the reason behind the absence of lag phase in the kinetics of BSA fibrillation process under the given condition.

### Kinetics of BSA fibrillation monitored from ThT fluorescence emission in the presence of osmolytes

**Fig 3:**
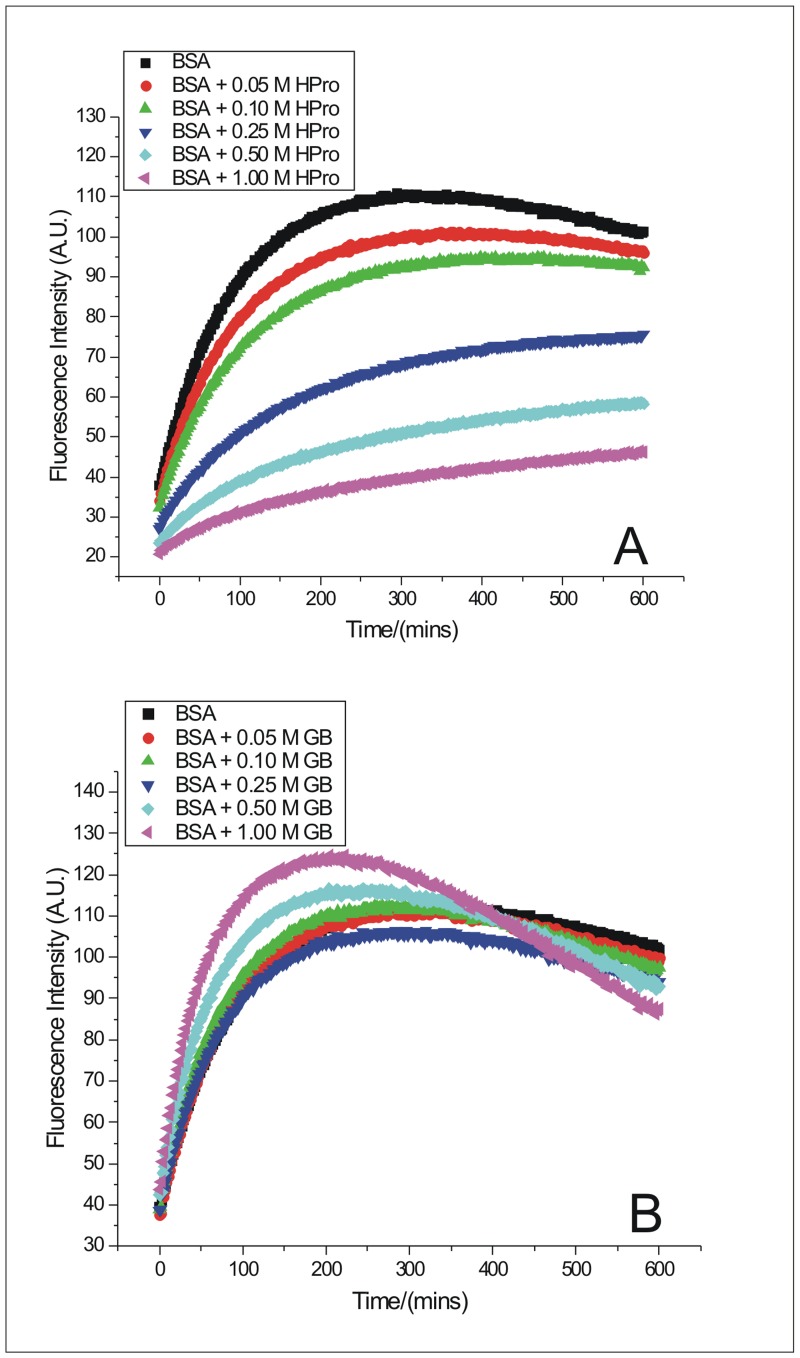
ThT binding assay in the presence of osmolytes. Kinetics of BSA fibrillation in the absence and in the presence of 0.05 M, 0.10 M, 0.25 M, 0.50 M and 1.00 M of osmolytes (A) 4-hydroxy-L-proline (HPro) and (B) Glycine Betaine (GB) monitored from the changes in fluorescence emission intensity of ThT as a function of time.

The fibrillation kinetics of 75.30 μM of BSA (pH 7.0) at 333.15 K, in the presence of 0.05 M, 0.10 M, 0.25 M, 0.50 M and 1.00 M proline (Pro), 4-hydroxy-trans-L-proline (HPro), glycine betaine (GB), sarcosine (Sarc), and sorbitol (Sorb) were monitored from fluorescence emission of ThT over a period of 600 minutes (Fig 3, S1 Fig). The results depict that with increasing concentration of the osmolytes, except GB, the extent of inhibition of BSA fibrillation also increases, evident from the reduction in ThT fluorescence emission. None of the osmolytes could induce a lag phase in the fibrillation process of BSA under the given condition and therefore, the onset of fibrillation of BSA remained same, in the absence and presence of various concentrations of these osmolytes. However, suppression of BSA fibrillation to different extent was achieved in presence of highest concentration (1.00 M) of the osmolytes proline, hydroxyroline, sarcosine and sorbitol, except glycine betaine. Hydroxyproline, at 1.00 M concentration, was found to be maximally efficient in suppressing the fibrillation process of BSA, followed by sorbitol, sarcosine and proline in their potency sequence which is—Hydroxyproline> Sorbitol> Sarcosine> Proline> Glycine betaine. The higher concentrations of GB (0.50 M and 1.00 M) seemed to affect negatively, by inducing further fibrillation of BSA molecules.

Shifting of t_1/2_ (the time point at which the fluorescence emission intensity of ThT is half the maximum signifying that 50% of fibrillation of the protein has taken place) towards the right along the time axis with change of physical conditions denote a decrease in the rate of the process in general. Similar shift in t_1/2_ of BSA fibrillation with increasing concentration of hydroxyproline, sorbitol, sarcosine and proline signifying a gradual decrease in the rate of BSA fibrillation with increasing osmolyte concentration, was observed (Fig 3, S1 Fig). It was also observed that at t = 0 minutes, the fluorescence emission of ThT decreased with increasing concentration of hydroxyproline (Fig 3A). Similar effect was seen for the other osmolytes as well, except glycine betaine, but to a smaller extent (Fig 3B, S1 Fig). Such an observation could be attributed to some minor conformational changes of the BSA molecules in the presence of increasing concentration of these osmolytes, that might have altered the ThT binding sites on BSA.

### Kinetics of BSA fibrillation monitored from Rayleigh scattering intensities in the presence of osmolytes

**Fig 4:**
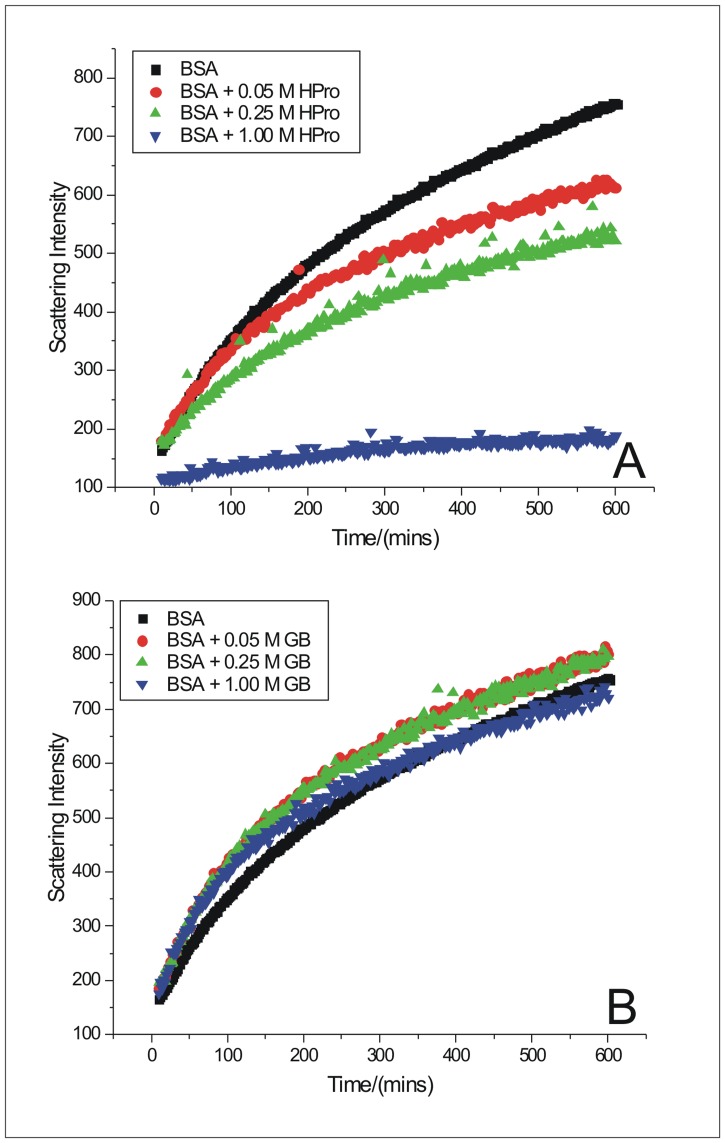
Rayleigh scattering measurements in the presence of osmolytes. Kinetics of BSA fibrillation in the absence and in the presence of 0.05 M, 0.25 M and 1.00 M of osmolytes (A) 4-hydroxy-L-proline (HPro) and (B) Glycine Betaine (GB) monitored from the Rayleigh Scattering Measurements as a function of time.

The measurements of scattered light intensity with time, in the spectrofluorimeter, for BSA molecules (75.30 μM) in the absence and presence of 0.05 M, 0.25 M, 1.00 M of Pro, HPro, GB, Sarc, and Sorb were done at 333.15 K temperature and pH 7.4 (Fig 4, S2 Fig). The increase in intensity over a period of 600 minutes denotes aggregation of BSA. In the presence of increasing concentration of the osmolytes, the Rayleigh scattering intensity of BSA aggregates decreased to different extent for different osmolytes, except for glycine betaine. This once again, suggests the inhibition of the aggregation process to differential extent, brought about by these osmolytes, under the given condition. The results of Rayleigh scattering experiments also show the absence of any lag phase in the kinetics of BSA aggregation and that none of the osmolytes could induce any lag phase in the process. Similar to the above ThT binding assay, the Rayleigh scattering experiments also show the same sequence of efficiency of these osmolytes at 1.00 M concentration, in bringing about inhibition of BSA fibrillation- Hydroxyproline> Sorbitol> Sarcosine> Proline> Glycine betaine. However, the difference in efficiencies of sorbitol and sarcosine seems to be very small (S2 Fig).

### Determination of kinetic parameters of fibrillation of BSA in the absence and presence of osmolytes

The kinetic traces of increase in the fluorescence emission of ThT, upon fibrillation of BSA in the absence and presence of the osmolytes at pH 7.4 and 333.15 K, were fitted to the following stretched exponential function [32, 41]

$$F=F^{\infty }+\Delta F\text{exp}[-{\left(kt\right)}^{n}]$$ (2)

where, *F* is observed fluorescence emission intensity of ThT at any time *t*, *F*^∞^ is the final fluorescence emission intensity of ThT, Δ*F* is the difference between the final and initial fluorescence emission intensities, *k* is the rate of fibrillation of BSA and *n* is any number that signifies cooperativity of the process. The results are shown in Table 1.

**Table 1:** Values of the kinetic parameters *n* and rate constant *k*, for fibrillation of BSA in the absence and presence 0.05 M, 0.10 M, 0.25 M, 0.50 M and 1.00 M of 4-hydroxy-trans-L-proline (HPro), sorbitol (Sorb), sarcosine (Sarc), proline (Pro), and glycine betaine (GB) at pH 7.4 and 333.15 K.

Solution	*n*	10^2^ *k*/min^-1^
Only BSA	1.076 ± 0.004	1.338 ± 0.004
BSA + 50mM HPro	1.034 ± 0.005	1.195 ± 0.004
BSA + 100mM HPro	1.045 ± 0.007	1.100 ± 0.004
BSA + 250mM HPro	1.091 ± 0.011	0.895 ± 0.005
BSA + 500mM HPro	1.101 ± 0.013	0.877 ± 0.006
BSA + 1 M HPro	1.074 ± 0.015	0.860 ± 0.007
BSA + 50mM Sorb	1.063 ± 0.005	1.356 ± 0.004
BSA + 100mM Sorb	1.081 ± 0.006	1.216 ± 0.004
BSA + 250mM Sorb	1.068 ± 0.007	1.071 ± 0.004
BSA + 500mM Sorb	1.091 ± 0.009	1.018 ± 0.005
BSA + 1M Sorb	1.088 ± 0.010	0.994 ± 0.005
BSA + 50mM Sarc	1.057 ± 0.006	1.210 ± 0.004
BSA + 100mM Sarc	1.032 ± 0.006	1.155 ± 0.004
BSA + 250mM Sarc	1.007 ± 0.007	1.085 ± 0.005
BSA + 500mM Sarc	1.028 ± 0.009	1.055 ± 0.005
BSA + 1 M Sarc	1.027 ± 0.010	0.997 ± 0.006
BSA + 50mM Pro	1.121 ± 0.004	1.319 ± 0.003
BSA + 100mM Pro	1.082 ± 0.006	1.214 ± 0.004
BSA + 250mM Pro	1.069 ± 0.005	1.219 ± 0.003
BSA + 500mM Pro	1.036 ± 0.006	1.226 ± 0.005
BSA + 1M Pro	1.072 ± 0.008	1.088 ± 0.005
BSA + 50mM GB	1.082 ± 0.005	1.342 ± 0.004
BSA + 100mM GB	1.080 ± 0.006	1.475 ± 0.005
BSA + 250mM GB	1.075 ± 0.005	1.402 ± 0.004
BSA + 500mM GB	1.098 ± 0.009	1.783 ± 0.008
BSA + 1M GB	1.177 ± 0.029	2.276 ± 0.032

Reported errors are the standard errors of fitting each data into the stretched exponential function *F*=*F*^∞^+Δ*F*exp[−(*kt*)^*n*^].

The rate of fibrillation of BSA, thus obtained, in the absence of any osmolyte is [(1.338 ± 0.004)·10^−2^] min^-1^. The rate of fibrillation of BSA gradually decreased from [(1.195 ± 0.004)·10^−2^] min^-1^ to [(0.860 ± 0.007)·10^−2^] min^-1^, [(1.356 ± 0.004)·10^−2^] min^-1^ to [(0.994 ± 0.005)·10^−2^] min^-1^, and [(1.210 ± 0.004)·10^−2^] min^-1^ to [(0.997 ± 0.006)·10^−2^] min^-1^ in the presence of increasing concentrations (0.05 M to 1.00 M) of HPro, Sorb and Sarc respectively (Table 1). The slightly higher value, [(1.356 ± 0.004)·10^−2^] min^-1^, obtained for rate of fibrillation of BSA in the presence of the lowest concentration (0.05 M) of Sorb compared to the value of [(1.338 ± 0.004)·10^−2^] min^-1^ obtained in the absence of any osmolyte, is in coherence with our observation of slightly higher intensity of ThT’s fluorescence emission during BSA fibrillation in the presence of 0.05 M of Sorb. In the presence of Pro (0.05 M to 1.00 M), the rate of fibrillation of BSA though decreased from [(1.319 ± 0.003)·10^−2^] min^-1^ to [(1.088 ± 0.005)·10^−2^] min^-1^ respectively, yet there was no trend observed at the intermediate concentrations of Pro (Table 1). The values of *k*, obtained for fibrillation of BSA in the presence of 0.05 M, 0.10 M, 0.25 M, 0.50 M and 1.00 M of GB were [(1.342 ± 0.004)·10^−2^] min^-1^, [(1.475 ± 0.005)·10^−2^] min^-1^, [(1.402 ± 0.004)·10^−2^] min^-1^, [(1.783 ± 0.008)·10^−2^] min^-1^, and [(2.276 ± 0.032)·10^−2^] min^-1^ respectively (Table 1). These values of *k* obtained in the presence of GB, once again supports the inefficiency of GB in inhibiting the fibrillation process of BSA under the given condition. The rate of fibrillation of BSA almost doubled in the presence of the maximum concentration of GB. The values of *k*, obtained for fibrillation of BSA in the presence of maximum concentration (1.00 M) of each of the osmolytes support the following order of efficiency of these osmolytes in inhibiting the fibrillation process of BSA- Hydroxyproline> Sorbitol> Sarcosine> Proline> Glycine betaine. The values *n* is around 1 in all the above cases. The above calculation of the kinetic parameters from the kinetic traces of Rayleigh Scattering was avoided due to presence of noise in the latter data. The dependences of the rate constant of the first order, on the concentration of chaperones can be used for estimation of the dissociation constant for chaperone-target protein complex [43].

### Transmission Electron Microscopy

**Fig 5:**
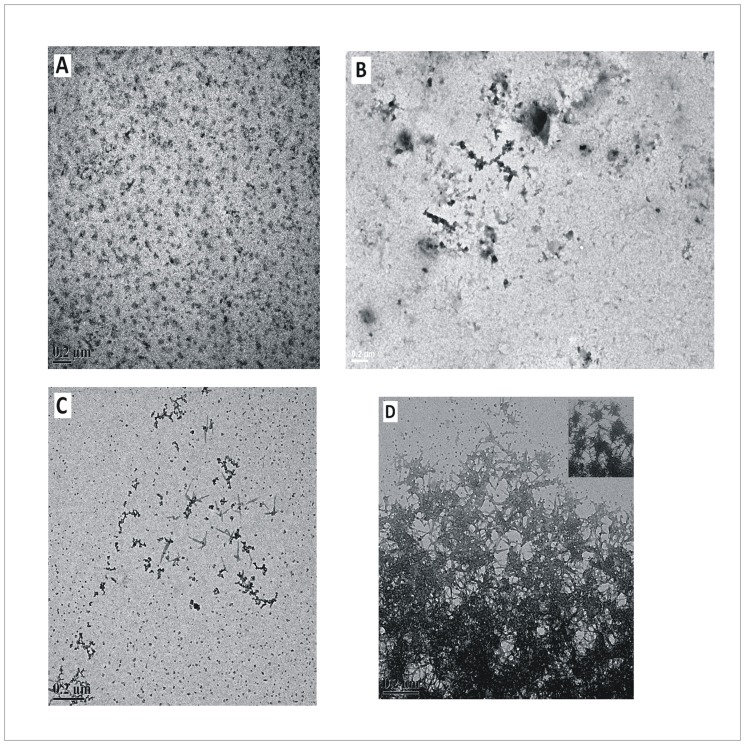
Transmission Electron Microscopy of native BSA and its stages of aggregation in the absence of osmolytes. Transmission Electron Microscopic images of BSA solution (A) in native state (t = 0 minutes), and after the incubation at 333.15 K for (B) 5 minutes, (C) 50 minutes (D) 600 minutes, respectively, in the absence of any osmolyte. Scale bar = 0.2 μm. The inset in (D) shows the magnified view (Scale bar = 100 nm).

**Fig 6:**
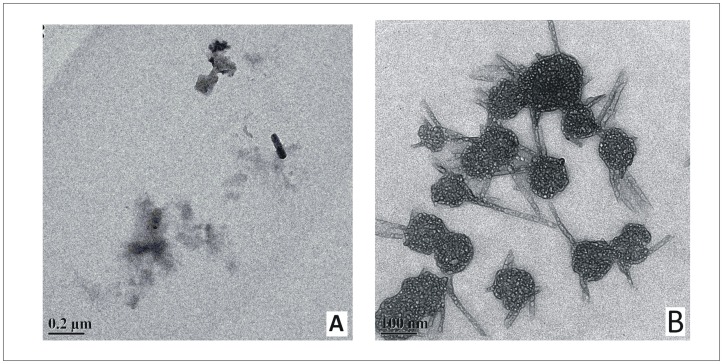
Transmission Electron Microscopy of BSA aggregates formed in the presence of osmolytes. Representative Transmission Electron Microscopic images of BSA solution after incubation at 333.15 K for a time period of 600 minutes, in the presence of 1.00 M concentration of (A) HPro and (B) GB. Scale bar = 0.2 μm and 100 nm for (A) and (B) respectively.

The TEM images of the different stages of BSA fibrillation occurring at 333.15 K temperature, in the absence and presence of 1.00 M concentration of the osmolytes, are shown in Figs 5 and 6 and supporting information (S3 Fig). The native state does not show any prominent oligomeric species (Fig 5A). Upon heating BSA solution at 333.15 K in the absence of any osmolyte, some nonfibrillar aggregates from association of monomers were observed (Fig 5B) at the first stage of aggregation after time t = 5 minutes. In the next stage after t = 50 minutes, these aggregates had started to take up fibrillar morphology forming small fibrils or protofibrils (Fig 5C). Finally, after a period of 600 minutes of the fibrillation process in the absence of any osmolyte, dense supramolecular network of fibrils of BSA was observed (Fig 5D).

Upon incubation of BSA at 333.15 K temperature in the presence of 1.00 M each of HPro, Sorb and Sarc for a period of 600 minutes, few non-fibrillar clumps/aggregates were observed (Fig 6A, S3A and S3B Fig). In case of the fibrillation process of BSA over a period of 600 minutes in presence of 1.00 M Pro, few small and discrete fibrillar bundles of BSA were observed (S3C Fig), supporting the weaker efficiency of Pro in inhibiting BSA fibrillation as observed from ThT fluorescence assay and Rayleigh scattering above. In the presence of GB, fibrillar/protofibrillar species were observed all over the field (Fig 6B) and this observation is in coherence with the inefficiency of GB in inhibiting BSA fibrillation under the given condition as observed from ThT Fluorescence Assay and Rayleigh Scattering Measurements, above.

### Dynamic Light Scattering

**Fig 7:**
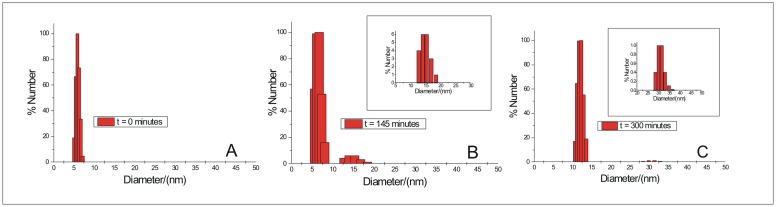
Size distribution of BSA molecules with the progress of aggregation from t = 0 to 300 minutes, in the absence of osmolyte, at pH 7.4. Representative size distributions at t = 0, 145 and 300 minutes are shown in (A), (B) and (C).

The size distribution of BSA molecules with the progress of their aggregation in the absence of any osmolyte was monitored at several time points from t = 0 to 300 minutes. The results are shown in Fig 7 and supporting information (S4 Fig). At t = 0, all molecules are in the native state showing a mean diameter around (5.9±0.21) nm (Fig 7A). After 5 minutes, i.e., at stage 1 of aggregation, the size increases slightly to about (6.8±0.20) nm (S4A Fig). This small increase in size could be due to the loosening up of the native conformation of the BSA molecules to yield partially unfolded states competent for advancement of the aggregation process. These species thus obtained after 5 minutes, are expected to be a combination of high reactive and low reactive unfolded forms [44]. There is a gradual build up of sizes of about 12.51 nm, 13.52 nm, 14.61 nm and 15.79 nm after 50 minutes (S4B Fig), indicating the formation of oligomers which increased in number after 145 minutes (Fig 7B). Two new peaks at 16.8 nm and 18.4 nm also appeared after t = 145 minutes (Fig 7B). However, the presence of the partially unfolded monomers of size distribution from (5.6–8.1) nm are still observed upto 145 minutes and these are majority in number (Fig 7B). This observation once again suggests the presence of unfolded forms of different rate of aggregation right from the initial stage of aggregation [44]. At t = 300 minutes, the mean diameter shifted to (12.2±0.16) nm with appearance of much smaller peaks around 30 nm (Fig 7C). The insets in Fig 7B and 7C show the enlarged views of the number percent of the minor contributors to the size distribution. After 300 minutes, no peak for any native or partially unfolded monomeric states or intermediates are observed signifying the presence of all oligomers only. This signifies that all monomers become a part of the oligomeric protofibrils at this stage leading to the saturation of fluorescence emission of ThT. These oligomers could be correlated to the small fibrils or protofibrils observed at the intermediate stage of BSA aggregation process (in absence of any osmolyte) from transmission electron microscopy (Fig 5C). At physiological pH, the formation of aggregates of BSA of sizes 15 nm, 17 nm, 22 nm and 28 nm at temperatures 338.15 K, 343.15 K, 348.15 K and 353.15 K respectively, has been reported from study on thermal aggregation of bovine serum albumin using asymmetrical flow field-flow fractionation [45].

**Fig 8:**
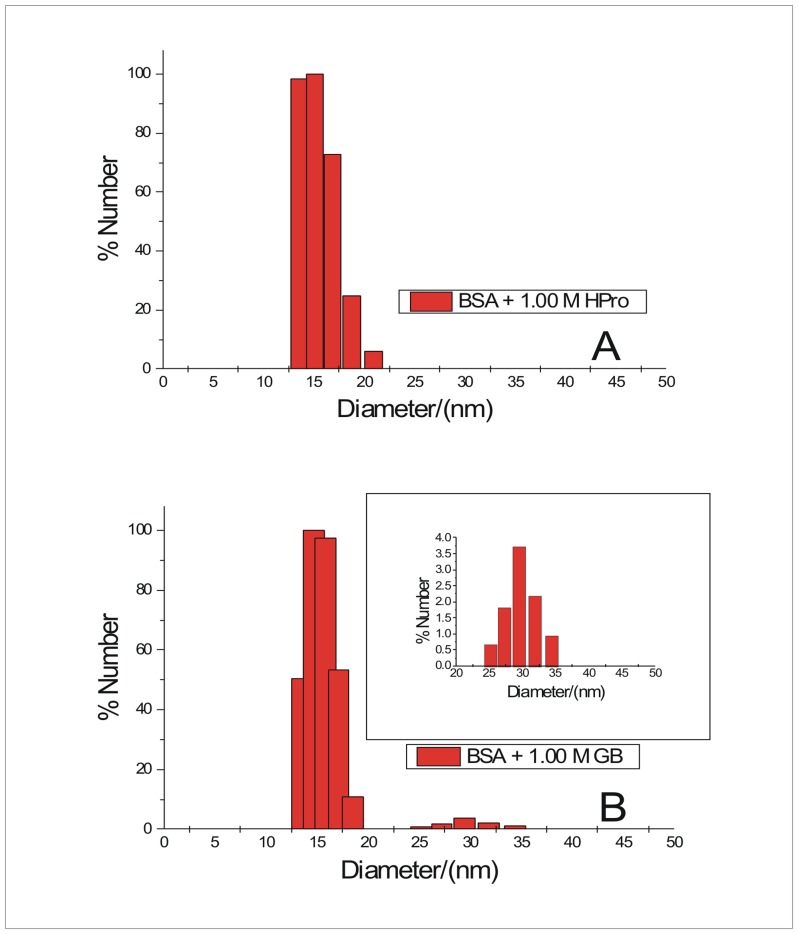
Size distribution of BSA aggregates formed in the presence of osmolytes upto t = 300 minutes. Representative size distribution of BSA aggregates formed in the presence of 1.00 M (A) Hpro and (B) GB

The size distribution obtained in the presence of the 1.00 M concentration of the osmolytes is given in Fig 8 and supporting information (S5 Fig). The mean diameter of the aggregates formed in the presence of 1.00 M hydroxyproline, sorbitol, sarcosine and proline are (15.4±0.33) nm, (15.4±0.26) nm, (11.4±0.30) nm, and (14.9±0.18) nm. These sizes denote the formation of small oligomers in the presence of these osmolytes. In the presence of 1.00 M glycine betaine major size distribution is around (14.7±0.21) nm with smaller peaks at 25.2 nm, 27.2 nm, 29.4 nm, 31.8 nm and 34.4 nm (Fig 8B). Therefore, the bigger oligomers or aggregates are still formed in the presence of glycine betaine similar to the case of size distribution obtained when BSA is allowed to fibrillate in the absence of any osmolyte. This observation also suggests the inefficiency of GB in inhibiting the fibrillation process of BSA under the given condition of heat denaturation. An extremely minor contribution from peaks at (36.4–46.6) nm diameter range is also observed when BSA is allowed to aggregate in the presence of 1.00 M Pro (S5C Fig, inset). The relative percentage of these bigger oligomers formed in the presence of Pro are comparatively much less than those in the presence GB.

Hence the results of the size distribution obtained for BSA aggregates formed in the absence and presence of the osmolytes, suggest that the formation of the bigger oligomers are crucial for advancement of the BSA fibrillation process to the mature stage that comprises of the dense fibrillar assembly as observed under the transmission electron microscope (Fig 5D). Also these bigger oligomers, soon after their formation, seem to get associated into the mature fibrillar assembly which probably settles down, evident from their relatively lower percentage compared to the smaller oligomers.

### ANS binding

**Fig 9:**
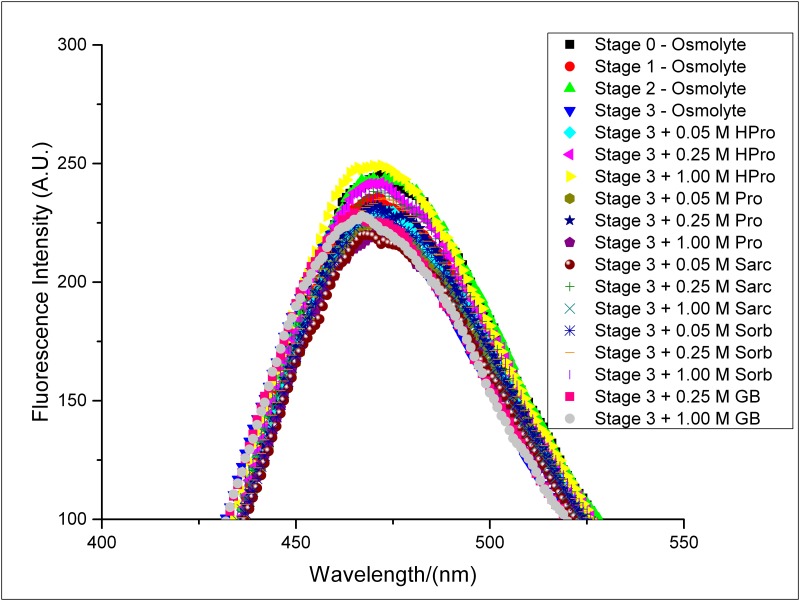
ANS binding. Fluorescence emission of ANS upon interaction with the different stages of BSA aggregates formed in the absence of osmolytes and final aggregates formed in the presence of different concentrations of these osmolytes- HPro, Sorb, Sarc, Pro and GB.

The fluorescence emission intensities of ANS dye upon interaction with native BSA and different stages of BSA aggregates formed in the absence of any osmolyte as well as the aggregates formed in the presence of certain concentrations of the osmolytes has been shown in Fig 9. Presence of five hydrophobic sites on BSA for binding of ANS at pH 7.0 has already been reported [46]. Our results show the absence of any significant change in the emission intensity and wavelength of maximum fluorescence emission of ANS when it binds to different stages of BSA aggregate formed in the absence of osmolytes and the aggregates formed in the presence of osmolytes. This suggests the absence of time dependent growth of any amorphous aggregates of BSA in the absence and presence of the osmolytes under our optimized condition of BSA aggregation. This supports the initial amorphous association of BSA monomers to form a certain nuclei, observed at the first stage of aggregation in the absence of any osmolyte, from Transmission Electron Microscopy (Fig 5B). In the second stage these nuclei show their structural reorganization to form protofibrillar structures and further fibrillar mesh downstream, as per our observation from Transmission Electron Microscopy (Fig 5C and 5D), thereby supporting the ANS binding results of no further increase in number of the amorphous structures.

### Mode of interaction of the fibrils with the osmolytes from ITC

**Fig 10:**
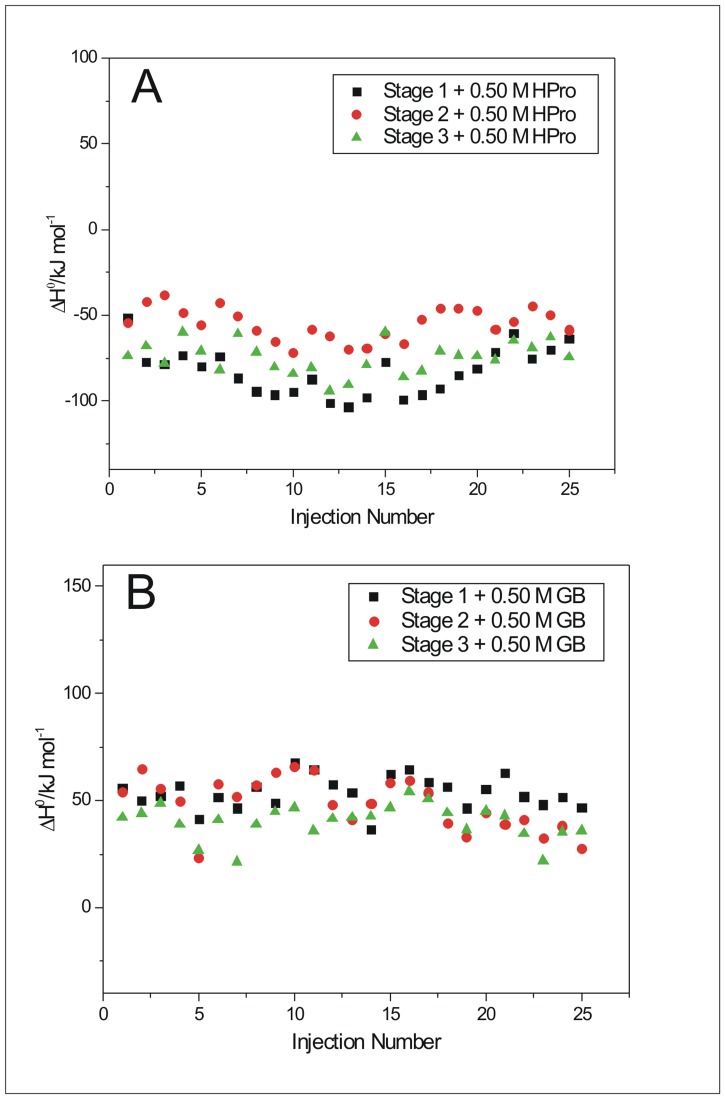
Isothermal Titration Calorimetry (ITC) experiments. Representative ITC profile for the titration of stages (1 to 3) of BSA aggregates into 0.50 M of (A) hydroxyproline (HPro) and (B) glycine betaine (GB).

The representative ITC profiles for titration of different stages of BSA aggregates into 0.50 M of each of the osmolyte solutions- HPro, Sorb, Sarc, Pro and GB- and after subtraction of the corresponding heats of dilution of the material in the syringe and cell, are shown in Fig 10 and supporting information (S6 Fig). All the titrations were carried out at 298.15 K. The stages of BSA aggregates, collected after different time (t) intervals of the fibrillation process, have been defined as stage 1, 2 and 3 obtained after t = 5 minutes, t = 50 minutes and t = 300 minutes of BSA fibrillation process. Separate experiments for titration of the stages of BSA aggregates into buffer and buffer into each of the osmolyte solutions of 0.50 M were carried out. The dilutions of the stages of BSA aggregates into buffer do not show any significant difference in the heats of dilutions (S6A Fig). The limiting standard enthalpies of interaction (Δ*H*°) between a particular stage of BSA aggregate and that of an osmolyte solution are summarized in Table 2. The values of (Δ*H*°) were obtained from either, linear fit of the data points in cases where the heat changes showed concentration/injection dependent trend or averaging the heat changes where no such trend was observed. The values of the limiting standard enthalpies of interaction (Δ*H*°) were all corrected for the effect of heats of dilutions of solutions in the syringe (BSA aggregates) as well as that in the cell (osmolyte). The values of (Δ*H*°) provide insights into the probable mode interaction and thus mechanism of inhibition of BSA aggregation process brought about by these osmolytes, under the given conditions. A representative thermogram, showing the raw data of injection of the stages (1 to 3) of BSA aggregation into 0.50 M HPro solution at pH 7.4 and 298.15 K is given in S7 Fig.

**Table 2:** Limiting standard enthalpies (Δ*H*°) of interaction (in kJ mol^-1^) of the different stages of BSA aggregates with 0.50 M concentration of hydroxyproline, sorbitol, sarcosine, proline and glycine betaine.

Osmolytes	Heat changes (kJ mol^-1^)		
	Stage 1	Stage 2	Stage 3
**Hydroxyproline**	- (83 ± 3)	- (55 ± 2)	- (72 ± 3)
**Sorbitol**	49 ± 3	99 ± 5	59 ± 5
**Sarcosine**	0.42 ± 0.99	- (12 ± 1)	- (8 ± 2)
**Proline**	121 ± 8	45 ± 9	40 ± 11
**Gycine Betaine**	54 ± 2	49 ± 2	40 ± 2

Reported errors are the standard errors of linear fitting of each of the dilution corrected data on heat of interaction.

Exothermic heat of interaction implying polar interactions between the osmolyte molecules and those of insulin and lysozyme, have been shown by our group [47,48] to play significant roles in prevention of fibrillation of the proteins. The exothermic heats of interaction obtained in this study when BSA fibrils were titrated into 0.50 M hydroxyproline could either be because of enhanced H-bonding in the hydration layer of the protein [49] or preferential interaction of these osmolytes with the solvent exposed polar moieties on native BSA molecules, thereby stabilizing its native form. Among the osmolytes used in this study, hydroxyproline showed maximum exothermicity and as well as maximum potency in efficient inhibition of BSA fibrillation. The extra hydroxyl group present in the hydroxyproline molecule when compared with the chemical structure of proline molecule (Fig 1A and 1D), could possibly contribute to the exothermicity. Out of the two substituents- a carboxyl and a hydroxyl group of the hydroxyproline molecule, one could interact with the surface charges on the protein while the other with the aqueous solvent, thereby stabilizing the native structure via this osmolyte mediated solvation. At the intermediate stage, as the protofibrils are formed, most of those interactions with the surface charges are probably lost, owing to the conformational rearrangement leading to protofibril formation. Hence there is a decrease in the exothermicity from Δ*H*° = {-(83 ± 3)} kJ mol^-1^ to {-(55 ± 2)} kJ mol^-1^ for interaction of hydroxyproline with stage 1 and stage 2, respectively (Table 2). There is again an increase in the heat of interaction yielding Δ*H*° = {-(72 ± 3)} kJ mol^-1^ for interaction of stage 3 with hydroxyproline. These, exothermic heats of interaction of hydroxyproline could also be contributed from its ability to interact with the polar moieties on the stage 2 and stage 3 aggregates of BSA molecules, thereby interfering with the salt bridges and H-bonding that are crucial within the fibril architecture and subsequently bringing about an extremely efficient inhibition of BSA fibrillation, compared to all the other osmolytes considered in our study. The above mentioned mode of interaction of hydroxyproline with the stage 1 aggregates of BSA in particular, will not be possible with proline since it lacks the extra hydroxyl group. This justifies the observed endothermic heats of interaction of proline with BSA (Table 2) when compared to those of hydroxyproline, inspite of the structural similarity between proline and hydroxyproline (Fig 1A and 1D).

The enthalpy values (Table 2), obtained for titration of the different stages of BSA fibrils into 0.50 M sorbitol are endothermic indicating hydrophobic interaction, yet sorbitol seems to be a potent inhibitor of BSA fibrillation evident from the ThT binding assay and Rayleigh Scattering. The mode of protein stabilization in the presence of sugars and polyols (sorbitol) have been shown to be by strengthening of pairwise hydrophobic interactions between hydrophobic groups in a protein molecule and this strengthening of hydrophobic interactions is in turn dependent on the changes in structure of water brought about by these polyols [50]. Extensive preferential hydration of the protein molecules in the presence of polyols would increase the tendency of the exposed non-polar groups to be buried in the protein interior thereby playing significant role in strengthening of hydrophobic interactions in the protein, with a resultant stabilization of the native structure of the protein [51, 52]. A similar mechanism of extensive preferential hydration of BSA molecules is assumed to be taking place in our system, in the presence of sorbitol thereby inhibiting the fibrillation of BSA significantly. The Δ*H*° value becomes more endothermic for the interaction of stage 2 aggregates with sorbitol (Δ*H*° = 99 ± 5 kJ mol^-1^) than that with stage 1 aggregates (Δ*H*° = 49 ± 3 kJ mol^-1^). Sugars and polyols, being polyhydroxy compounds, are themselves extremely hydrophilic. Therefore, they will have unfavourable interactions with the non-polar/hydrophobic moieties of the cosolute (protein here) in the same solution, thereby driving the hydrophobic moieties away from the polyols and water. This explains and supports the above noted increase in endothermic heat of interaction of sorbitol with stage 2 aggregates of BSA which are expected to have many partially folded BSA conformations with exposed hydrophobic moieties. Again the endothermic heat of interaction decreases to (Δ*H*° = 59 ± 5 kJ mol^-1^) for interaction of stage 3 BSA aggregates with sorbitol. This suggests a dominance of the exothermic effect of H-bonding of water due to enhanced structuring of the solvent brought about by sorbitol combined with a decrease in the extent of intramolecular hydrophobic interactions of the BSA molecules at stage 3 of aggregation. As observed from DLS, stage 3 aggregates of BSA do not show any peak for monomeric species which imply presence of all fibrillar species of BSA at this stage and these oligomeric species are resistant to further conformational changes, in the presence of sorbitol. Therefore sorbitol will not be capable of reverting the fibrillar species back to native BSA and exerts its major inhibitory action at the initial and intermediate stages of BSA aggregation.

Direct interaction of osmolytes with the protein backbone leads to stabilization of the denatured state [53]. In the presence of osmolyte, the thermodynamic stability of a protein will be influenced by two effects- (i) preferential hydration of protein thereby exclusion of the osmolyte molecules from the protein surface and destabilization due to interaction of non-polar moieties of the osmolyte molecules with the exposed hydrophobic amino acid side chains of the protein [54]. The latter effect seems to dominate in the presence of Glycine betaine supported by the obtained endothermicity from our ITC results (Table 2), where the methyl groups from GB (Fig 1E) are expected to favourably interact with the exposed hydrophobic parts of the protein at 333.15 K. The endothermic heat of interaction of GB is maximum with stage 1 of BSA aggregation (Δ*H*° = 54 ± 2 kJ mol^-1^) compared to the heats of interaction with stages 2 (Δ*H*° = 49 ± 2 kJ mol^-1^) and 3 (Δ*H*° = 40 ± 2 kJ mol^-1^) of BSA aggregation. This decrease in the endothermic heat of interaction of GB with the advanced stages of BSA aggregates could be due to alteration of GB binding sites on BSA molecules as the latter already forms proto/prefibrils and subsequent fibril network. This observation provides evidence that the major driving force for BSA aggregation in the presence of GB, is the destabilization of the native and the stabilization of stage 1 aggregates via hydrophobic interaction. These stage 1 morphologies are competent enough to give rise to protofibrils which in turn are responsible for formation of the final fibrillar network of BSA molecules under the given conditions of aggregation. Glycine betaine, though extremely potent in stabilizing native conformation of proteins associated with Photosystem II complex of cyanobacteria and plants [55], however, induced aggregation and precipitation of GST-GFP fusion protein [56]. L.R. Singh *et al*. showed pH dependent different effect of GB on different proteins, where RNase-A was stabilized under different pH conditions, lysozyme stability remained unaffected from pH 5–2 while α-LA was destabilized below pH 5 in presence of GB [57].

However sorbitol has not been potent enough to prevent insulin fibrillation, despite the hydrophobic heat of interaction of insulin with sorbitol, as reported by our group previously [47]. These observations for GB and sorbitol, suggests protein specific action of osmolytes [53].

The endothermic mode of interaction of proline with stage 1 (Δ*H*° = 121 ± 8 kJ mol^-1^) suggests a combination of strengthening of pairwise intramolecular hydrophobic interactions similar to that of sorbitol as well as direct interaction of proline with the hydrophobic moieties of the protein. Hence observed the highest endothermic value (Δ*H*° = 121 ± 8 kJ mol^-1^) when compared to all the other heats of interaction obtained for all the osmolytes considered. Interaction of proline with protein backbone or exposed hydrophobic moieties will decrease the stability of the protein and increase its propensity to denature and aggregate, similar to our discussion for interaction of glycine betaine above. This observation for proline is in coherence with the relative potencies of the osmolytes considered in inhibiting the aggregation process (Figs 3 and 4 and S1 and S2 Figs), where efficiency of proline stands after hydroxyproline, sorbitol and sarcosine. The above endothermicity however decreases significantly to Δ*H*° = (45 ± 9) kJ mol^-1^ and Δ*H*° = (40 ± 11) kJ mol^-1^ for interaction of stage 2 and 3 with proline. Alternately the above increase in exothermicity suggests the occurrence of polar or electrostatic interactions between these stages and the zwitterionic proline molecules. This further suggests that proline is capable of inhibiting the fibrillation process of BSA by disrupting the polar or salt bridge interactions or even interfering with the H-bonding moieties of the β-sheets of the fibrils. Electrostatic interactions have been shown to play an important role to confer stability to insulin fibrils [58] and Aβ oligomers [59].

Investigation using molecular dynamics simulation by our group [60], has shown that sarcosine enhances the hydrogen bonded tetrahedral structure of water. This will contribute to an exothermic heat of interaction. However the small endothermic heat of interaction of stage 1 with sarcosine (Δ*H*° = 0.42 ± 0.99 kJ mol^-1^) suggests a mixed contribution from exothermicity due structuring of water, endothermicity due to increased burial of nonpolar moieties leading to hydrophobic effect and some direct interactions of sarcosine molecules with the exposed hydrophobic regions on BSA, with a predominance of the latter two. Sarcosine was observed to be intermediate in its efficiency among the osmolytes studied here, in inhibiting BSA aggregation (Figs 3 and 4 and S1 and S2 Figs) and thereby supports the occurrence of its direct interaction with the hydrophobic moieties on BSA leading to a decrease in stability of BSA. The exothermic heats of interaction of sarcosine with stages 2 {Δ*H*° = -(12 ± 1)} kJ mol^-1^ and 3 {(Δ*H*° = -(8 ± 2)} kJ mol^-1^ of BSA aggregates provide evidence for interaction of sarcosine with the essential polar/ion groups/H-bond donors or acceptors which are expected to contribute to building up of the stable protofibril and mature fibrillar structure of BSA aggregates.

### Discussion on the possible mode of aggregation of BSA and its inhibition by the various osmolytes

Based on our results of TEM and DLS, we propose upon heating, loosening of the native structure leads to increased exposure of the otherwise buried hydrophobic patches which drives association of monomers to form certain short lived nuclei. This association of monomers is devoid of any lag as observed from the kinetics of BSA fibrillation monitored by time dependent Rayleigh Scattering and fluorescence emission of ThT (Figs 3 and 4 and S1 and S2 Figs). Absence of lag phase in the kinetics of BSA fibrillation under the given condition has been explained under section “Kinetics of BSA fibrillation monitored from ThT fluorescence emission in presence of osmolytes”. These nuclei thus obtained, are formed from the partially unfolded structures. Now in order to avoid repulsion of the like charges on the surface, the monomer units of the nuclei quickly undergo conformational rearrangement to form small fibrillar morphologies evident from the TEM image of the intermediate stage of BSA aggregation (Fig 5C) [3]. No time delay was observed in the above step of BSA fibrillation, evident from immediate rise in ThT’s fluorescence emission. The basal level of fluorescence emission intensity of ThT in presence of the native BSA (Fig 2B) increases with accumulation of these fibrillar forms. Each of these small fibrillar structures or protofibrils associate with one another to give rise to the supramolecular fibril network obtained at the final stage of BSA aggregation seen under transmission electron microscopy (Fig 5D). Correlation of DLS results at t = 300 minutes to the TEM images of the fully mature BSA fibrils, signifies the settling down of these heavier supramolecular fibril assemblies. Only the small fibril assemblies or protofibrils made of dimers and about five monomers remain in the solution to yield DLS data. Association of protofibrils into the final fibril network are expected to start at the intermediate stages themselves, yielding a mixture of the above morphologies at the intermediate stages with a predominance of the protofibrillar species though. Though fluorescence emission of ThT reaches a saturation after 300 minutes which remains steady upto the 600^th^ minute but the existing protofibrils are expected to continue giving rise to the fibril network. This can be confirmed from Rayleigh scattering as well, which does not show saturation upto 600 minutes. This conclusion is also supported by the results of DLS and TEM. Absence of any significant difference in the fluorescence emission intensity and the wavelength of fluorescence emission of the dye ANS, in the presence of the different stages of BSA aggregates obtained in the absence of any osmolyte, denote the absence of growth of any non-fibrillar or amorphous morphology during the period of BSA fibrillation.

Now the osmolyte inhibitors can act at any of these stages of BSA fibrillation by any kind of interaction thereby reducing/inhibiting the process of fibrillation. As far as our study is concerned, the osmolytes were added to the native BSA which was then allowed to aggregate under the given conditions. Therefore, in the presence of the osmolytes, the inhibition of protein aggregation can be achieved by any of the following means- (a) stabilization of the native structure of the protein thereby delaying or completely abolishing any conformational change that could potentially lead towards protein aggregation, (b) can interact with any of the morphologies or assemblies formed during the fibrillation process, such as the initial multimeric nuclei, the protofibrillar structures and the supramolecular fibrillar mesh/network. Modes of interaction with these intermediates, obtained in the process of fibrillation, also seem to vary for the different osmolytes used leading to different extent of inhibition. Among the osmolytes studied here, hydroxyproline was observed to be capable of directly interacting with the surface charges of BSA molecules via polar interactions, thereby preventing further unfolding of the protein to expose the buried hydrophobic amino acids to the aqueous solvent under the given condition of heat denaturation. This is evident from the exothermic heat of interaction of hydroxyproline with the stage 1 aggregates of BSA where the protein molecules were still in monomeric stage as observed from DLS. Hence hydroxyproline is expected to exert its major inhibitory effect via type (a) kind of inhibition discussed above leading to stabilization of the native structure of BSA. On the other hand type (b) kind of inhibition mechanism has been observed in presence hydroxyproline, proline as well as sarcosine, which we have discussed in detail in the discussion section of ITC results. The exothermic heats of interaction of these three osmolytes with the BSA fibrillation intermediates obtained from ITC suggest the significance of the polar interactions that are crucial within the fibril architecture and that interference with or disruption of these polar interactions by the osmolyte molecules is leading to inhibition/reduction of further fibrillation of BSA under the given condition of heat denaturation. These polar interactions necessary for stable fibril architecture could be the characteristic H-bonding of the β-sheets of the fibrils or electrostatic interactions of the opposite charges on amino acid residues, etc. However mechanism of inhibition of BSA fibrillation by sorbitol was observed to be different from the other three. Sorbitol mainly carried out its inhibitory effect by affecting the structure of water leading to extensive preferential hydration of BSA molecules which resulted in increased burial of the nonpolar/hydrophobic moieties of the protein. This also suggests that the major inhibitory action of sorbitol occurs after t = 0 minutes of the heat denaturation process when the partially folded BSA monomers and oligomers with exposed hydrophobic patches, accumulate, which is during the intermediate stages of aggregation. However, GB has been completely inefficient in inhibiting the fibrillation of BSA owing to its direct interaction with the hydrophobic moieties of the protein backbone thereby stabilizing the aggregation competent nuclei/oligomers of BSA.

The inhibitory actions of these osmolytes (HPro, Sorb, Sarc, Pro) though reduced the extent of fibrillation of BSA to different degrees yet a certain intermediate nuclei in the path of aggregation was still formed even in the presence of the highest concentration of these osmolytes. This is supported by our observations from ThT Binding Assay, Rayleigh Scattering Measurements, DLS and TEM. In case of fluorescence emission of ThT and Rayleigh Scattering Measurements, the intensity at t = 600 minutes was higher than that at t = 0 minute (Figs 3 and 4 and S1 and S2 Figs) which signifies the presence of some non native species after the given period of BSA fibrillation in the presence of 1.00 M concentration of these osmolytes. DLS results also show the presence of oligomeric species with no peak for the monomer, after 600 minutes of fibrillation process of BSA in the presence of 1.00 M concentration of these four osmolytes. This observation was further validated from the TEM images which show the presence some small clumps/non fibrillar oligomeric assemblies formed after 600 minutes of aggregation of BSA in the presence of 1.00 M of HPro, Sorb and Sarco. This suggests that the aggregation/fibrillation of BSA was arrested at an intermediate stage in the presence of these osmolytes. However, in case of BSA aggregation in the presence of 1.00 M concentration of Pro, some small and discrete fibrillar bundles of BSA were observed under Transmission Electron Microscopy. This observation is in coherence with the results of ThT binding assay and Rayleigh Scattering measurements, which showed lesser efficiency of Pro in comparison with HPro, Sorbi and Sarco, to inhibit the process of BSA fibrillation. In case of BSA fibrillation in the presence of GB, all our results from the various experimental techniques such as ThT Binding Assay, Rayleigh Scattering Measurements, DLS and TEM support the inefficiency of GB in inhibiting BSA fibrillation under the given condition.

## Conclusions

Our study suggests that not only hydrophobic effect but also polar interactions play important role in the fibrillation process of BSA at 333.15 K under physiological pH. In the presence of osmolytes, combination of preferential exclusion leading to hydration of the protein molecules and direct interaction of the osmolyte molecules with the protein backbone are expected to take place with a predominance of one over the other, which would determine the ultimate stability of the protein molecules. Also under conditions of temperature (T < T_m_) selected for our study, the action of these chemical chaperones or osmolytes on the aggregation process will be determined by their effects on the unfolding stage [61]. That osmolyte which would stabilize the native structure maximally via favourable interactions with the exposed polar/ionic side chains on protein molecules, is expected to bring about the inhibition of fibrillation with maximum efficiency (eg: HPro in this study). However, the ability of the osmolytes to interferre with or disrupt the interoligomer interactions crucial for fibrillar growth would also lead to inhibition of the fibrillation process (eg: HPro, Pro and Sarc in this study). Sorbitol exerted its major inhibitory action by the mode of extensive preferential hydration of the protein molecules which was maximum with the intermediate stage of BSA aggregates thus formed and suggests that this mode of inhibition of fibrillation will be maximally effective in the presence of the intermediates having partially exposed hydrophobic patches. However, preferential interaction of osmolyte with the exposed hydrophobic patches would be detrimental, leading to stabilization of aggregation competent nuclei (eg: GB in our case). We observed the relative efficiencies of the osmolytes in bringing about the inhibition of BSA fibrillation to be- Hydroxyproline> Sorbitol> Sarcosine> Proline> Glycine betaine, from ThT binding assay, Rayleigh Scattering measurements and calculation of the relative rates of BSA fibrillation. Rayleigh Scattering when compared to DLS gave a real time monitoring of the fibrillation process of BSA. Correlating our observations with those from literature it can be concluded that the inhibitory action of the osmolytes is protein specific (eg.: Sorbitol and GB) discussed already. Though we used stretched exponential function for calculation of the kinetic parameters yet simple exponential function could be used for the same since denaturation of the protein proceeds as a reaction of the first order with the parameter *n* being close to unity [62]. The above study on the BSA fibrillation mechanism and the different modes of inhibition of the various osmolytes would guide the development of drug targets against protein fibrillation.

## Supporting information

S1 Fig ThT binding assay. Kinetics of BSA fibrillation in absence and in presence of 0.05 M, 0.10 M, 0.25 M, 0.50 M and 1.00 M of osmolytes (A) sorbitol (Sorb), (B) sarcosine (Sarc) and (C) proline (Pro) monitored from the changes in fluorescence emission intensity of ThT as a function of time.
(TIF)

S2 Fig Rayleigh scattering measurements. Kinetics of BSA fibrillation in absence and in presence of 0.05 M, 0.25 M and 1.00 M of osmolytes (A) sorbitol (Sorb), (B) sarcosine (Sarc) and (C) proline (Pro) monitored from the Rayleigh Scattering Measurements as a function of time.
(TIF)

S3 Fig Transmission Electron Microscopic images of BSA aggregates formed in presence of osmolytes. Transmission Electron Microscopic images of BSA solution after incubation at 333.15 K for a time period of 600 minutes, in presence of 1.00 M concentration of (A) sorbitol (Sorb), (B) sarcosine (Sarc) and (C) proline (Pro). Scale bar = 0.2 μm for (A) and (B) and 100 nm for (C).
(TIF)

S4 Fig Size distribution of BSA molecules with the progress of aggregation in absence of osmolyte, at pH 7.4. Size distributions at (A) t = 5 minutes and (B) t = 50 minutes.
(TIF)

S5 Fig Size distribution of BSA molecules with the progress of aggregation in presence of osmolyte, at pH 7.4. Size distribution of BSA aggregates formed in presence of 1.00 M (A) sorbitol (Sorb), (B) sarcosine (Sarc) and (C) proline (Pro).
(TIF)

S6 Fig Isothermal Titration Calorimetry (ITC) experiments. ITC profiles for the titration of stages (1 to 3) of BSA aggregates into (A) buffer, and into 0.50 M of (B) sorbitol (Sorb), (C) sarcosine (Sarc) and (D) proline (Pro), at pH 7.4.
(TIF)

S7 Fig Heat flow versus time. Representative ITC thermogram showing the raw data of injection of (A) stage 1, (B) stage 2 and (C) stage 3 of BSA aggregates into 0.50 M HPro solution at pH 7.4.
(TIF)
