# Supplementary figures and images for: Selective inhibition of aggregation/fibrillation of bovine serum albumin by osmolytes: Mechanistic and energetics insights
Source: PLoS One. 2017 Feb 16;12(2):e0172208. doi: 10.1371/journal.pone.0172208 (PMC5312929; doi:10.1371/journal.pone.0172208)

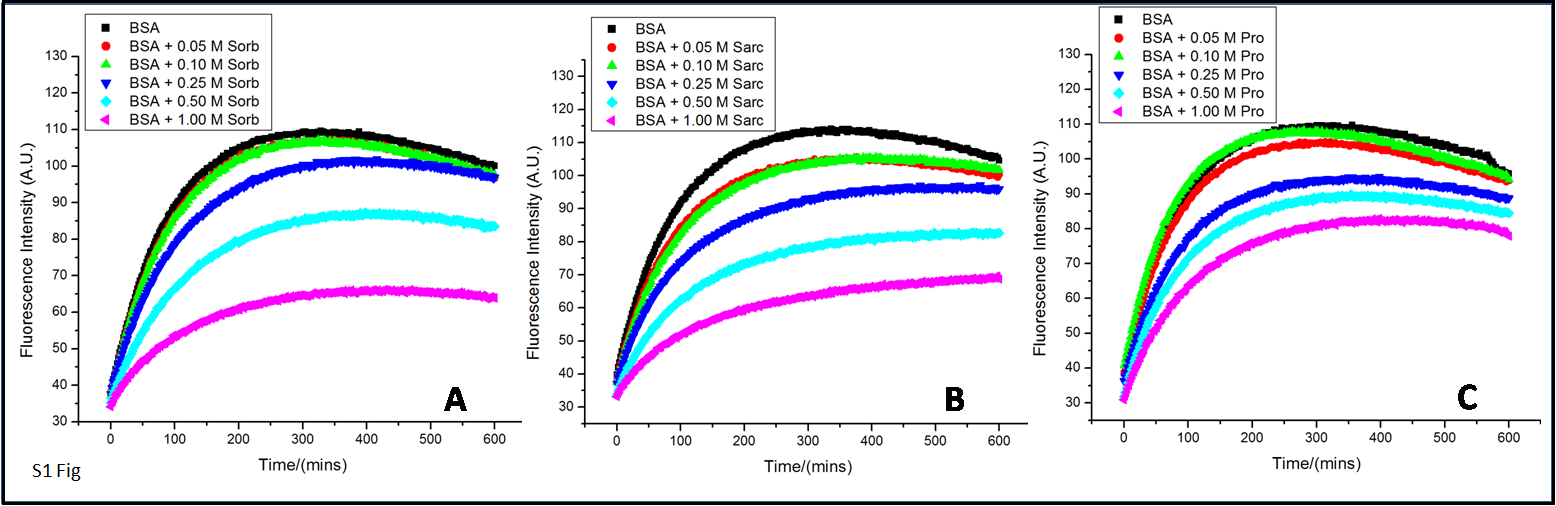

Supplement: S1 Fig — Kinetics of BSA fibrillation in absence and in presence of 0.05 M, 0.10 M, 0.25 M, 0.50 M and 1.00 M of osmolytes (A) sorbitol (Sorb), (B) sarcosine (Sarc) and (C) proline (Pro) monitored from the changes in fluorescence emission intensity of ThT as a function of time. (TIF) [file pone.0172208.s001.tif]

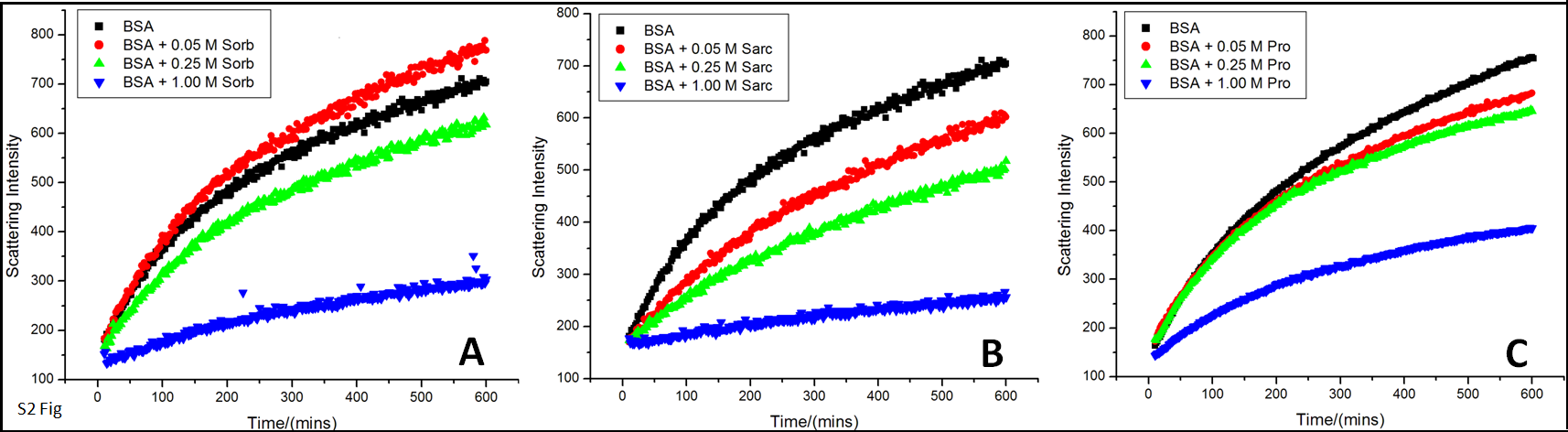

Supplement: S2 Fig — Kinetics of BSA fibrillation in absence and in presence of 0.05 M, 0.25 M and 1.00 M of osmolytes (A) sorbitol (Sorb), (B) sarcosine (Sarc) and (C) proline (Pro) monitored from the Rayleigh Scattering Measurements as a function of time. (TIF) [file pone.0172208.s002.tif]

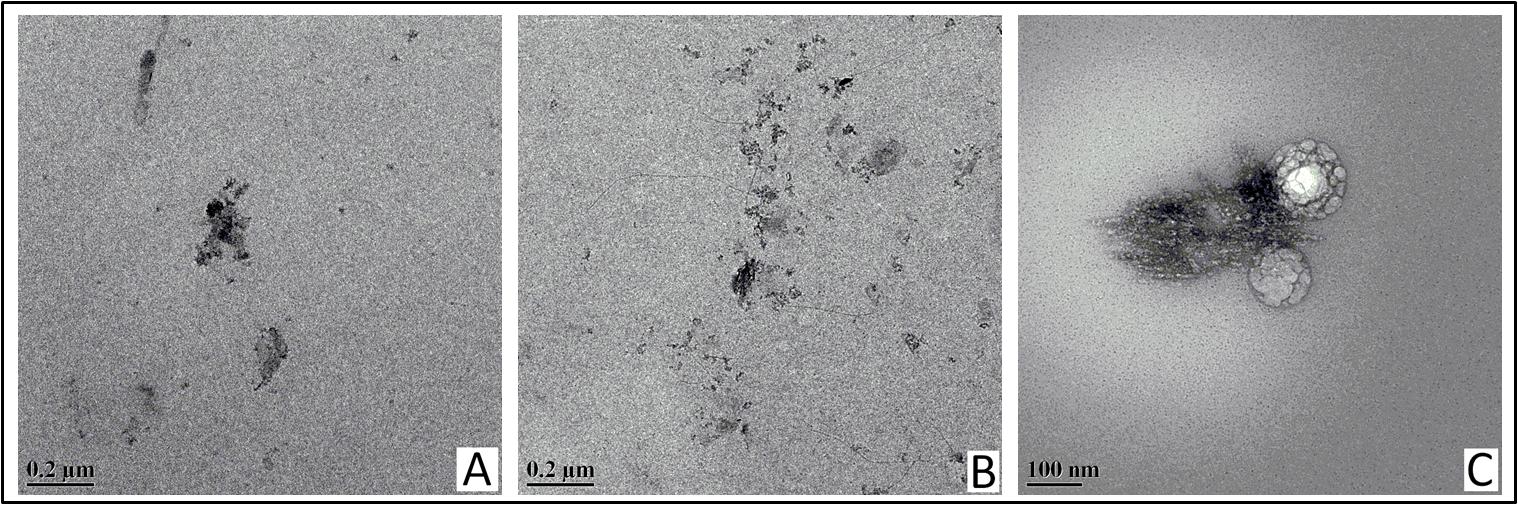

Supplement: S3 Fig — Transmission Electron Microscopic images of BSA solution after incubation at 333.15 K for a time period of 600 minutes, in presence of 1.00 M concentration of (A) sorbitol (Sorb), (B) sarcosine (Sarc) and (C) proline (Pro). Scale bar = 0.2 μm for (A) and (B) and 100 nm for (C). (TIF) [file pone.0172208.s003.tif]

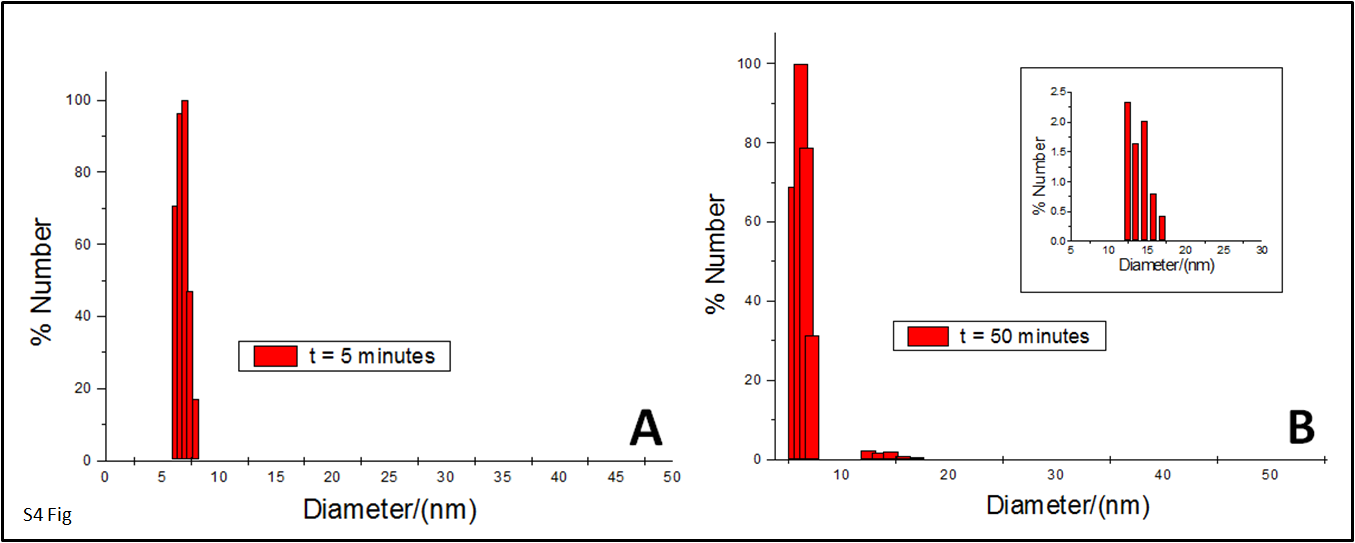

Supplement: S4 Fig — Size distributions at (A) t = 5 minutes and (B) t = 50 minutes. (TIF) [file pone.0172208.s004.tif]

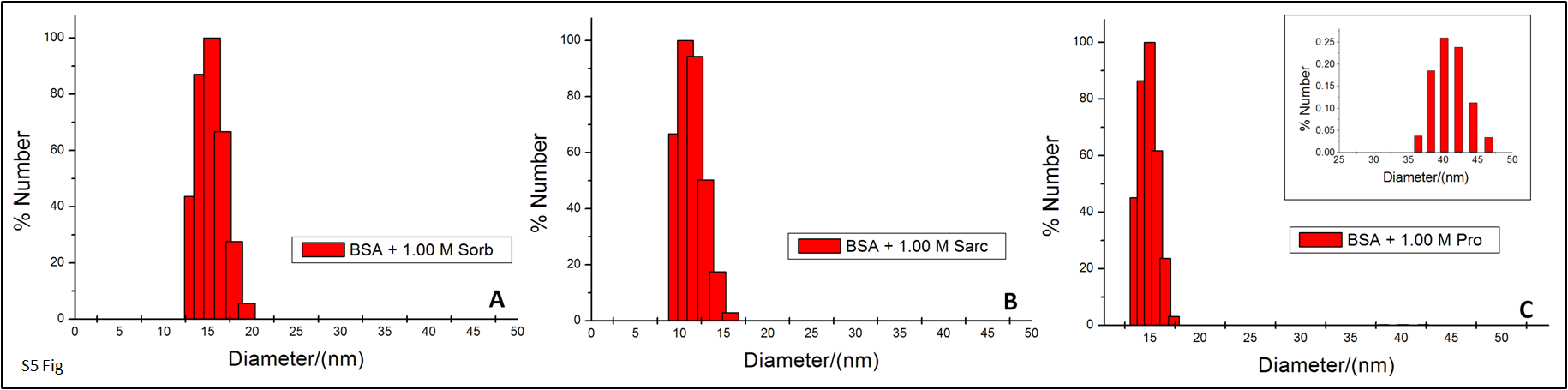

Supplement: S5 Fig — Size distribution of BSA aggregates formed in presence of 1.00 M (A) sorbitol (Sorb), (B) sarcosine (Sarc) and (C) proline (Pro). (TIF) [file pone.0172208.s005.tif]

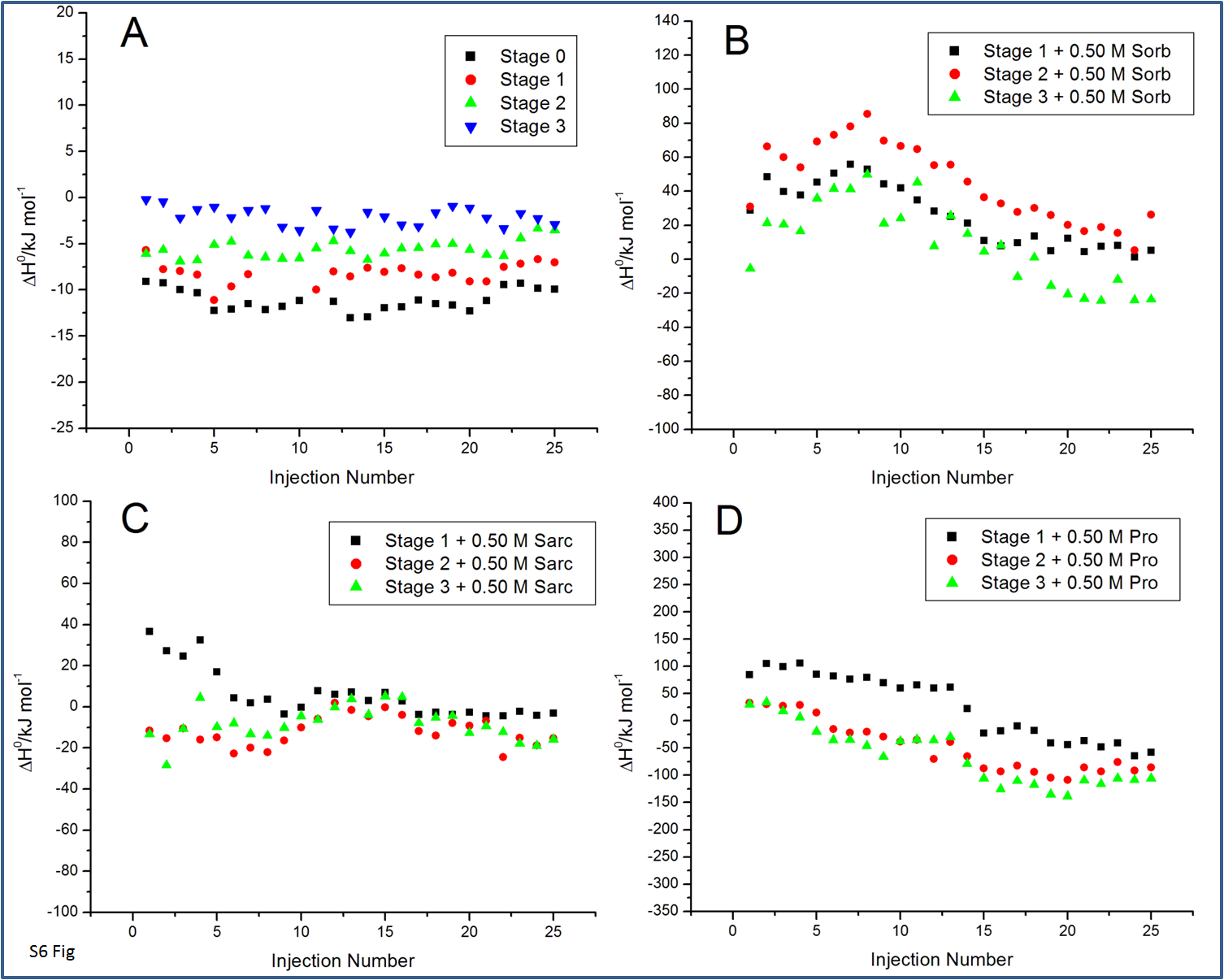

Supplement: S6 Fig — ITC profiles for the titration of stages (1 to 3) of BSA aggregates into (A) buffer, and into 0.50 M of (B) sorbitol (Sorb), (C) sarcosine (Sarc) and (D) proline (Pro), at pH 7.4. (TIF) [file pone.0172208.s006.tif]

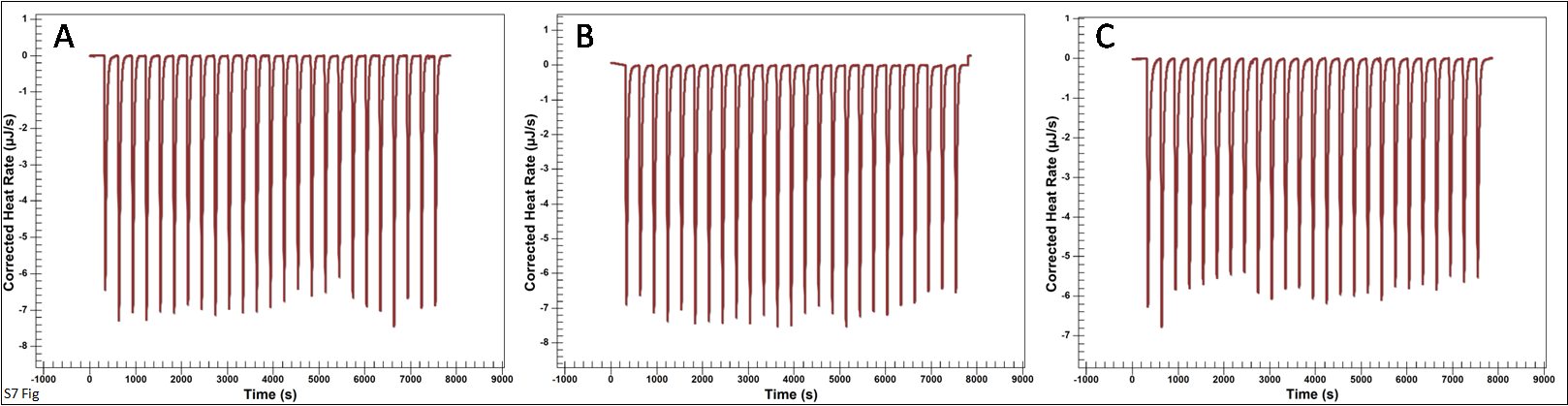

Supplement: S7 Fig — Representative ITC thermogram showing the raw data of injection of (A) stage 1, (B) stage 2 and (C) stage 3 of BSA aggregates into 0.50 M HPro solution at pH 7.4. (TIF) [file pone.0172208.s007.tif]
